# Risk factors for surgical site infection after lumbar laminectomy and/or discectomy for degenerative diseases in adults: A prospective multicenter surveillance study with registry of 4027 cases

**DOI:** 10.1371/journal.pone.0205539

**Published:** 2018-10-16

**Authors:** Satoshi Ogihara, Takashi Yamazaki, Hirohiko Inanami, Hiroyuki Oka, Toru Maruyama, Kota Miyoshi, Yuichi Takano, Hirotaka Chikuda, Seiichi Azuma, Naohiro Kawamura, Kiyofumi Yamakawa, Nobuhiro Hara, Yasushi Oshima, Jiro Morii, Rentaro Okazaki, Yujiro Takeshita, Sakae Tanaka, Kazuo Saita

**Affiliations:** 1 Department of Orthopaedic Surgery, Saitama Medical Center, Saitama Medical University, Saitama, Japan; 2 Department of Orthopaedic Surgery, Musashino Red Cross Hospital, Tokyo, Japan; 3 Department of Orthopaedic Surgery, Inanami Spine and Joint Hospital, Tokyo, Japan; 4 Department of Medical Research and Management for Musculoskeletal Pain, 22nd Century Medical and Research Center, Faculty of Medicine, University of Tokyo, Tokyo, Japan; 5 Department of Orthopaedic Surgery, Saitama Rehabilitation Center, Saitama, Japan; 6 Department of Orthopaedic Surgery, Yokohama Rosai Hospital, Kanagawa, Japan; 7 Department of Orthopaedic Surgery, Iwai Orthopaedic Medical Hospital, Tokyo, Japan; 8 Department of Orthopaedic Surgery, Faculty of Medicine, Gunma University, Gunma, Japan; 9 Department of Orthopaedic Surgery, Saitama Red Cross Hospital, Saitama, Japan; 10 Department of Spine and Orthopaedic Surgery, Japanese Red Cross Medical Center, Tokyo, Japan; 11 Department of Orthopaedic Surgery, Tokyo Metropolitan Komagome Hospital, Tokyo, Japan; 12 Department of Orthopaedic Surgery, Faculty of Medicine, University of Tokyo, Tokyo, Japan; 13 Department of Orthopaedic Surgery, Sanraku Hospital, Tokyo, Japan; George Washington University, UNITED STATES

## Abstract

Surgical site infection (SSI) is a significant complication after spinal surgery and is associated with increased hospital length of stay, high healthcare costs, and poor patient outcomes. Accurate identification of risk factors is essential to develop strategies to prevent wound infections. The aim of this prospective multicenter study was to determine the independent factors associated with SSI in posterior lumbar surgeries without fusion (laminectomy and/or herniotomy) for degenerative diseases in adult patients. From July 2010 to June 2014, we conducted a prospective multicenter surveillance study in adult patients who developed SSI after undergoing lumbar laminectomy and/or discectomy in ten participating hospitals. Detailed patient and operative characteristics were prospectively recorded using a standardized data collection format. SSI was based on the Centers for Disease Control and Prevention definition. A total of 4027 consecutive adult patients were enrolled, of which 26 (0.65%) developed postoperative SSI. Multivariate regression analysis indicated two independent factors. An operating time >2 h (P = 0.0095) was a statistically significant independent risk factor, whereas endoscopic tubular surgery (P = 0.040) was a significant independent protective factor. Identification of these associated factors may contribute to surgeons’ awareness of the risk factors for SSI and could help counsel the patients on the risks associated with lumbar laminectomy and/or discectomy. Furthermore, this study’s findings could be used to develop protocols to decrease SSI risk. To the best of our knowledge, this is the first prospective multicenter study that identified endoscopic tubular surgery as an independent protective factor against SSI after lumbar posterior surgery without fusion.

## Introduction

Surgical site infection (SSI) after spinal surgery, which occurs in 0.7–12% of patients, is one of the most serious complications and could result in high morbidity, high mortality, poor operative outcomes, and increased healthcare costs [1, 2]. Thus, a variety of risk factors for SSI after spinal surgery have been previously identified to prevent this significant complication. These risk factors include advanced age [3], male sex [4], obesity [5, 6], previous spinal surgery [5], malnutrition [3], diabetes [5–7], smoking [5], spinal trauma [8, 9], corticosteroid use [5, 10], spinal instrumentation [11], posterior surgical approach [2], tumor resection [2], surgery involving sacrum [11], dural tear [12], conventional open spinal surgery instead of endoscopic tubular surgery [13, 14], increased estimated blood loss [7], and prolonged operating time [11, 15]. However, some of these studies were performed retrospectively at individual institutions and/or were limited by their relatively small sample size. Nevertheless, even in studies with a large sample size, the research method using nationwide database was proven inadequate for examining patient characteristics and individual pre- or intraoperative factors in detail.

Hence, well-designed studies, which are based on a prospective design and a large sample size, are needed to identify precise independent risk factors for SSI after spinal surgery. A multivariate analysis should also be performed to adjust for multiple associated factors within individual patients. In addition, standardized, hospital-based, multicenter surveillance methods utilizing the standard definition of SSI have been considered useful in determing risk factors [16–18].

Therefore, the aim of this study was to identify independent factors associated with SSI after posterior lumbar decompressive surgeries without fusion (laminectomy and/or discectomy) for degenerative diseases. In this study, we employed a prospective multicenter surveillance research method; prospectively collected multicenter data from a registry of more than 4000 patients were used.

## Materials and methods

### Data source

From July 2010 to June 2014, a prospective surveillance study of SSI following posterior lumbar surgeries without fusion (laminectomy and/or discectomy) for degenerative diseases in adult patients was conducted in ten participating Japanese hospitals. Detailed preoperative and intraoperative data on patient demographics, medical comorbidities, surgical procedures, and adverse events were recorded postoperatively using a standardized data collection format. Each patient was followed up for a minimum of 1 month. We excluded patients younger than 18 years old and those who underwent fusion surgery (with or without spinal instrumentation), percutaneous surgery, or open surgery for other conditions, such as infection, tumor, and trauma. This study was approved by the institutional review boards of Saitama Medical University, Musashino Red Cross Hospital, the University of Tokyo, Yokohama Rosai Hospital, Saitama Red Cross Hospital, Japanese Red Cross Medical Center, Tokyo Metropolitan Komagome Hospital, Sanraku Hospital, Sagamihara National Hospital, and Iwai Orthopedic Medical Hospital. With the observational nature of this study, the institutional review boards of the participating hospitals waived the need for consent from individuals. The opt-out information is available here: http://www.kawagoe.saitama-med.ac.jp/chiken/hec/. The collected patient data were anonymized and de-identified prior to analyses.

### Identification of SSI

In this study, SSI was defined based on the Centers for Disease Control and Prevention definition [19]. Briefly, a superficial SSI was defined as an infection involving the skin or subcutaneous tissue only occurring within 30 days after the operation. A deep SSI was defined as an infection that occurs within 30 days after the operation (if no implant was left in place) or within 1 year (if the implant was left in place), appears to be operation related, and involves deep soft tissues. Moreover, a deep SSI was further characterized by the presence of one or more of the following [19]: (1) purulent drainage from the deep incision; (2) a deep incision that spontaneously dehisces or is deliberately opened by a surgeon when the patient has at least one of the following signs or symptoms: fever (>38°C), localized pain, or tenderness, unless the site is culture-negative; (3) an abscess or other evidence of infection involving the deep incision that is found on direct examination, during reoperation, or by a histopathologic or radiologic examination; and (4) diagnosis of a deep incisional SSI by a surgeon or attending physician.

Microbiological culture results of all of the patients who have developed SSI were collected and recorded. In patients who underwent open debridement, microbiological cultures were taken to confirm the presence of SSI and to decide further treatment.

### Study measures

At each study hospital, the medical records of eligible adult patients who had undergone posterior lumbar spinal procedures without fusion for degenerative diseases were prospectively collected using standardized patient charts. The following patient and operative data were recorded: age at time of surgery, sex, height, weight, smoking status, diabetes mellitus, body mass index, American Society of Anesthesiologists score [20], surgical history in the operated area, steroid use, anatomic location of the surgery (lumbar spine only or including the sacrum [L5/S1 level]), type of operative procedure (with or without discectomy), emergency surgery, dural tear, endoscopic tubular surgery, use of an operative microscope, operating time, and intraoperative bleeding.

### Statistical analysis

We analyzed the relationship between SSI and potential risk factors. Student’s t-test was used to compare the means of the continuous variables between the SSI and non-SSI groups. For categorical variables, Pearson’s chi-squared test was used to assess the differences in the proportions between the two groups. Relative risks and 95% confidence intervals were calculated using univariate and multivariate logistic regression analyses. Because of the relatively low number of patients with SSI in this study, propensity score adjustment was performed for each of the risk factors in multivariable modeling.

Propensity score adjustment preserved the statistical power by reducing covariates into a single variable. A propensity score was created using binary logistic regression to provide predicted probability of SSI as a function of the important factors (univariate logistic regression analysis, *P*<0.20). For example, of eight explanatory variables, one was used as an explanatory variable and the remaining seven were used in the propensity score adjustment. Statistical analysis was performed using SPSS Statistics version 24 (IBM Corporation, Armonk, NY) and JMP Pro 14 (SAS Institute Japan, Tokyo, Japan). A *P* value of 0.05 was considered statistically significant.

## Results

From July 2010 to June 2014, a total of 4027 consecutive patients (female 1235, male 2792; mean age 59.2 years; age range 18–94 years) in ten Japanese hospitals were enrolled. The demographic characteristics of the patients included in this study are shown in Table 1.

**Table 1:** Demographic characteristics of the SSI and Non-SSI groups.

Characteristic	SSI group (n = 26)	Non-SSI group (n = 4001)	*P* value
Age (years), mean±SD	61.3±12.9	59.1±18.1	0.41
Male sex, n (%)	23 (88.5)	2769 (69.2)	0.034
Body mass index (kg/m^2^)	25.4±3.7	24.2±3.7	0.10
ASA score ≥2, n (%)	20 (76.9)	2492 (62.3)	0.13
Diabetes mellitus, n (%)	5 (19.2)	452 (11.3)	0.20
Hemodialysis, n (%)	0 (0.0)	49 (1.2)	0.57
Smoking, n (%)	8 (30.8)	717 (17.9)	0.089
Steroid use, n (%)	1 (3.8)	42 (1.0)	0.17
Discectomy	6 (23.1)	1667 (41.7)	0.055
Anatomic location of the surgery, n (%)			
Operation including L5/S1 level	33 (8.7)	947 (4.3)	0.91
Revision surgery	0 (0.0)	376 (9.4)	0.10
Dural tear	3 (11.5)	253 (6.3)	0.28
Endoscopic tubular surgery	4 (15.4)	1587 (39.7)	0.012
Use of an operative microscope	2 (7.7)	191 (4.8)	0.49
Use of a bio-clean room	6 (23.1)	1136 (28.4)	0.55
Emergency surgery	1 (3.8)	52(1.3)	0.26
Operative time (>2 h), n (%)	16 (61.5)	1249 (31.2)	0.0009
Intraoperative bleeding (ml)	174.0±264.2	126.8±230.4	0.30
Prophylactic intravenous administration of CEZ	26 (100.0)	3923 (98.1)	0.47

SSI, surgical site infection; SD, standard deviation; ASA, American Society of Anesthesiologists; CEZ, cefazolin

The total incidence of SSI after surgery was 0.65% (26 cases; 17 deep SSI, 9 superficial SSI). With respect to the demographic characteristics, male sex, use of an endoscope, and an operative time >2 h were significantly associated with SSI (*P* < 0.05), while a higher body mass index, ASA score ≥2, smoking, steroid use, laminectomy, and revision surgery were correlated (*P* < 0.20) with SSI. These results are similar to those obtained with the univariate logistic regression analysis, except for revision surgery (Table 2).

**Table 2:** Univariate logistic regression analysis for SSI after lumbar laminectomy and/or herniotomy.

Characteristic	OR (95% CI)	*P* value
Age	1.01 (0.99–1.03)	0.549
Male sex	3.41 (1.02–11.38)	0.046
Body mass index	1.08 (0.99–1.18)	0.094
ASA score ≥2	2.02 (0.35–5.04)	0.13
Diabetes mellitus	1.87 (0.70–4.98)	0.21
Smoking	2.04 (0.88–4.70)	0.096
Steroid use	3.77 (0.50–28.47)	0.20
Discectomy	0.42 (0.17–1.05)	0.063
Operation including L5/S1 level	0.89 (0.12–6.57)	0.91
Dural tear	1.93 (0.58–6.48)	0.29
Endoscopic tubular surgery	0.28 (0.095–0.80)	0.018
Use of an operative microscope	1.66 (0.39–7.09)	0.49
Use of a bio-clean room	0.76 (0.30–1.89)	0.55
Emergency surgery	3.04 (0.40–22.84)	0.28
Operative time (>2 h)	3.53 (1.60–7.79)	0.0018
Intraoperative bleeding (ml)	1.00 (1.00–1.001)	0.29

SSI, surgical site infection; OR, odds ratio; CI, confidence interval; ASA, American Society of Anesthesiologists; CEZ, cefazolin. The odds ratio could not be calculated for variables that are 0 or 100% in any group; thus, no data for hemodialysis, revision surgery, prophylactic intravenous administration of CEZ) are included in the table.

Table 3 shows the results of the multivariate logistic regression analysis with propensity score adjustment, including the variables that correlated with SSI (*P* < 0.20) in the univariate logistic regression analysis.

**Table 3:** Multivariable Logistic regression analysis with propensity score adjustment for SSI after lumbar laminectomy and/or herniotomy.

Characteristic	OR (95% CI)	*P* value
Male sex	2.99 (0.89–10.0)	0.08
Body mass index	0.94 (0.86–1.03)	0.18
ASA score ≥2	1.63 (0.64–4.14)	0.30
Steroid use	4.20 (0.55–32.0)	0.17
Smoking	2.27 (0.97–5.30)	0.058
Discectomy	0.46 (0.18–1.16)	0.10
Endoscopic tubular surgery	0.33 (0.11–0.96)	0.040
Operative time (>2 h)	2.92 (1.30–6.55)	0.0095

SSI, surgical site infection; OR, odds ratio; CI, confidence interval; ASA, American Society of Anesthesiologists

The results suggested that an operative time >2 h (*P* = 0.0095, OR = 2.92, 95% CI 1.30–6.55) is an independent risk factor for SSI, while the use of an endoscope (*P* = 0.040, OR = 0.33, 95% CI 0.11–0.96) is an independent protective factor against SSI.

Microbiological cultures were routinely obtained in all 26 patients who developed SSI. Of the 26 patients, 21 (80.8%) underwent open debridement and 23 (88.5%) had a positive culture. No organisms were isolated in three of the 17 patients who developed deep SSI. Organisms were isolated in all nine patients who developed superficial SSI. Of the 23 patients with positive cultures, 22 (95.7%) had a single organism isolated and only one (4.3%) demonstrated polymicrobacterial growth (*Staphylococcus epidermidis* + *Pseudomonas aeruginosa*). *Staphylococcus aureus* was present in 65.2% (15/23) of the positive cultures; 46.7% (7/15) of these isolates demonstrated methicillin-resistant *S*. *aureus*. Coagulase-negative *Staphylococcus* was the next most common organism, which occurred in 21.7% (5/23) of the positive cultures (including the case of polymicrobial growth); methicillin resistance was noted in 60.0% (3/5) of the patients with coagulase-negative *Staphylococcus* (Table 4).

**Table 4:** Microbiological characteristics of SSI.

Organism(s)	No. of cases
*Staphylococcus aureus*	8
MRSA	7
Methicillin-resistant CNS	3
CNS	1
CNS *+ Pseudomonas aeruginosa*	1
*AB Streptococcus*	1
*Streptococcus equisimilis*	1
*Serratia marcescens*	1
Unknown	3

CNS, coagulase-negative staphylococci; MRSA, methicillin-resistant *Staphylococcus aureus*; SSI, surgical site infection.

## Discussion

Using a prospective multicenter surveillance research method, we identified the independent associated factors in adult patients who have developed SSI after posterior lumbar spinal surgeries without fusion (laminectomy and/or discectomy) for degenerative diseases.

An operating time >2 h was an independent risk factor (*P* = 0.0095) for postoperative SSI after adjusting for all other variables. This result is consistent with those in previous literature that reported a prolonged surgical procedure as a significant risk factor for SSI [11, 13, 21, 22]. Based on this finding, efforts to shorten the operative time could be useful in reducing SSI incidence. Ahn et al. reported that a longer operating time increases the risk for bacterial contamination in surgical wounds [23]. Moreover, dilution of bacteria by frequent intraoperative saline irrigation is reported to be effective in reducing bacterial contamination in the operated field, thereby preventing critical levels of infection [9]. In case of prolonged surgical procedure, Owen et al. described that repeated irrigation from an early stage of surgery is especially vital to reduce the number of bacteria in the operative wound [24]. Longer operative times also result in prolonged tissue retraction and thus tissue ischemia, necrosis, and desiccation. Hence, frequently releasing the tension on self-retractors could minimize tissue ischemia and necrosis due to intraoperative wound retraction during longer operative procedures [15].

Endoscopic tubular surgery was found to be a significant protective factor for SSI compared to conventional open laminectomy and/or discectomy in the multivariate analysis. Endoscopic tubular spinal surgery was developed as a minimally invasive surgery (MIS) for spinal diseases and has a relatively short history. Foley and Smith first described microendoscopic discectomy in 1997 as MIS for the treatment of lumbar disc herniation [25]; since then, endoscopic tubular surgery has been applied for the treatment of lumbar degenerative disorders, which was associated with favorable outcomes [26–30]. Microendoscopic laminectomy is one of the popular endoscopic surgical procedures; it is a modification of microendoscopic bilateral decompression via a unilateral approach for the treatment of lumbar spinal canal stenosis [30].

Some studies have demonstrated that endoscopic tubular surgery is associated with less invasiveness and lower SSI incidence compared with posterior open lumbar surgery, with comparable clinical outcomes [27, 29]. However, these studies involved a small number of patients from single institutions; thus, whether endoscopic surgery is indeed associated with lower SSI risk compared with open surgeries remains to be clarified.

Two recent retrospective studies with large sample sizes using Diagnosis Procedure Combination (DPC) database, which is a nationwide inpatient database in Japan, showed that endoscopic tubular surgery is associated with a lower SSI incidence compared with open decompressive surgeries without fusion [13, 14]. However, although these studies were persuasive and had large sample sizes, they had some disadvantages in clinical data collection. First, the database did not include any information after discharge; thus, SSI incidence was possibly under-reported. Second, the coded diagnoses may be less accurate than the diagnoses recorded in clinical researches because the code of DPC was made not only for the purpose of collecting data for clinical survey but also, originally, for insurance claims of hospitals. Therefore, the diagnosis was possibly coded with bias toward insurance billing. Furthermore, the database did not include detailed clinical information on risk factors for SSI as reported in previous literature, such as steroid use [5, 10], dural tear [12], surgery involving sacrum [11], revision surgery [5], and emergency surgery [31]. A retrospective study using a nationwide database is considered inferior to a prospective study in terms of clinical data collection accuracy. Nevertheless, despite the clinical importance of evaluating the risk for SSI, there is a paucity of studies that examined the difference in SSI incidence between open surgery and endoscopic tubular surgery through a prospective multicenter research method with a large sample size, which is partially because the history of endoscopic spinal surgery is relatively short.

In this study, the SSI incidence in adult patients who underwent endoscopic tubular surgery was almost threefold lower than that in patients treated with open laminectomy and/or discectomy (odds ratio, 0.33). In addition, data in this study are consistent with those in previous reports comparing the SSI incidence following minimally invasive spinal procedures with that after open surgeries [13, 14, 26]. To the best of our knowledge, this is the first prospective multicenter study that identified endoscopic tubular surgery as an independent protective factor for SSI after lumbar posterior surgery without fusion.

Endoscopic tubular surgery was developed with the concept of preserving posterior structures (e.g., skin, subcutaneous fat, and paravertebral muscles), and a smaller surgical field requirement; thus, the incised soft tissues and dead space are both reduced [32]. In addition, endoscopic surgery reduces the exposure of deeper tissues to the environment; only the field at the base of the tubular retractor is exposed, thereby reducing the possible contamination of the surface area [33]. The reduction in tissue trauma and in surgical field exposure could be associated with the lower SSI incidence following endoscopic tubular surgery.

This study has several limitations. First, the sample size of patients with infection was relatively small (n = 26). In contrast to previous research on SSI, which generally focused on a wide variety of spinal procedures (e.g., instrumented fusion surgery) and diseases (e.g., spinal trauma and spinal tumor) [2, 8, 9], this study included only the patients with SSI following specific types of procedures and diseases (i.e., lumbar laminectomy and/or herniotomy for degenerative diseases) without metal implants. Second, this study did not account for several factors, including experience level of the surgeon [34] and malnutrition [3], which were described as risk factors for SSI in some studies. Although the measured confounders were adjusted by a multivariate analysis, the results may still be biased because of unmeasured confounders. In addition, selection bias during patient enrollment was possible; however, we made an effort to minimize this bias by enrolling consecutive patients from multiple centers and not from a single center. The strengths of this study are the relatively large number of surgical procedures. In addition, the prospective multicenter surveillance design enabled a detailed study of the potential risk factors for SSI and identified independent risk factors for SSI after spinal surgery using multivariate logistic regression.

## Conclusions

In conclusion, we identified that an operative time >2 h is an independent risk factor for and endoscopic tubular surgery was an independent protective factor against SSI following lumbar laminectomy and/or herniotomy for degenerative diseases in adult patients. Surgeons’ awareness of these risk factors could help prevent SSI following surgery, and surgeons could explain the associated risks and complications to patients preoperatively. Moreover, the risk factors for SSI identified in this study may be used to design protocols that could reduce SSI incidence.

## Supporting information

S1 File Supporting information. Dataset of this study.
(XLS)
